# High-Intensity Exercise Improves Fatigue, Sleep, and Mood in Patients With Axial Spondyloarthritis: Secondary Analysis of a Randomized Controlled Trial

**DOI:** 10.1093/ptj/pzaa086

**Published:** 2020-05-04

**Authors:** Silje Halvorsen Sveaas, Hanne Dagfinrud, Inger Jorid Berg, Sella Arrestad Provan, Melissa Woll Johansen, Elisabeth Pedersen, Annelie Bilberg

**Affiliations:** 1 Department of Rheumatology, Norwegian National Advisory Unit on Rehabilitation in Rheumatology, Diakonhjemmet Hospital, Diakonveien 12, Oslo, Norway; 2 Department of Rheumatology, Norwegian National Advisory Unit on Rehabilitation in Rheumatology, Diakonhjemmet Hospital; 3 Department of Rheumatology, Diakonhjemmet Hospital; 5 Department of Physiotherapy, Martina Hansens Hospital, Bærum, Norway; 6 Department of Physiotherapy, University Hospital of North Norway, Tromsø, Norway; 7 Institute of Neuroscience and Physiology, Section of Health and Rehabilitation, Physiotherapy, Sahlgrenska Academy, University of Gothenburg, Gothenburg, Sweden

## Abstract

**Objective:**

Although exercise is recommended in the treatment of axial spondyloarthritis (axSpa), the focus has been on flexibility, and the effect of high-intensity exercises is unknown. The purpose of this study was to investigate the effect of high-intensity exercises on fatigue, sleep, and mood in patients with axSpA.

**Methods:**

In this secondary analysis of a randomized controlled trial, participants were recruited from outpatient clinics at 4 hospitals in Scandinavia. A total of 100 patients with axSpA were randomized to either an exercise group (n = 50) or a control group (n = 50). High-intensity exercise was provided 3 times per week for 3 months and supervised by a physical therapist. The controls received no intervention. Measurements were self-reported at baseline, 3 months, and 12 months: fatigue, using the Fatigue Severity Scale (range = 0–7, 7 = worst, ≥5 = severe); vitality, using the RAND 36-item short-form health survey (SF-36, range = 0–100, 100 = best); sleep, using the Pittsburgh Sleep Quality Index (range = 0–21, 21 = worst, >5 = poor quality); mood, using the General Health Questionnaire 12 (range = 0–36, 36 = worst); and general health, using the EUROQoL (range = 0–100, 100 = best).

**Results:**

A total of 38 participants (76%) in the exercise group followed ≥80% of the exercise protocol. At 3 months, there was a significant beneficial effect on fatigue (mean group differences = −0.4, 95% CI = −0.7 to −0.1), vitality (5.0, 95% CI = 1.1 to 10.5), mood (−2, 95% CI = −3.7 to −0.04), and general health (9.0, 95% CI = 3.3 to 14.7) but no effect on sleep (−1.1, 95% CI = −2.1 to 0.2). Compared with the control group, the exercise group had a reduced rate of severe fatigue and poor sleep. No differences were seen between the groups at 12 months.

**Conclusions:**

A 3-month exercise program had a beneficial effect on fatigue, sleep, mood, and general health in patients with axSpA at the end of the intervention; however, no long-term effects were seen.

**Impact:**

High-intensity cardiorespiratory and strength exercises should be considered as important in exercise programs for patients with axSpA.

Axial spondyloarthritis (axSpA) is a chronic inflammatory rheumatic disease primarily affecting the axial skeleton with a prevalence of 0.3% to 1.4%.^1^ AxSpA covers both patients with radiographic, structural damage of the axial skeleton (ankylosing spondylitis) and patients without structural damage (non-radiographic axSpA). The disease usually debuts in early adulthood, and men and women are equally affected by axSpA,^1^ but more men develop ankylosing spondylitis. The typical clinical features are back pain and stiffness, but patients report that besides pain, fatigue and sleep are their most important health aspects.^2^^,^^3^ Disease-related fatigue is described as a feeling of abnormal lack of energy that is not relieved by rest and unlike the feeling of tiredness in healthy adults.^4^ Fatigue is explained by an interplay between different factors such as inflammation, medication, pain, sleep, and deconditioning^5^ and is reported to correlate with mood disorders such as depression and anxiety.^6^ Hence, effective treatment strategies to reduce fatigue in patients with axSpA are needed.

Physical therapy with exercises is recommended as a cornerstone in the management of axSpA together with pharmacological treatment.^7^ Pharmacological treatment options (biologic agents) have been shown to be effective in reducing disease activity^8^ and overall sleep scores,^9^ but whether medication has a beneficial effect on fatigue and mood disorders is more uncertain.^10^ As in the general population, few patients with axSpA meet the general physical activity recommendations,^11^^,^^12^ and few practice vigorous^13^^,^^14^ and aerobic physical activity.^12^ Therefore, it may be hypothesized that an improvement in physical fitness may reduce the burden of fatigue in patients with axSpA.

Exercise is shown to have a positive effect on fatigue, vitality, mood,^15^^,^^16^ and sleep quality^17^ in the general population. The evidence for a similar effect in patients with axSpA is limited as fatigue, sleep, and mood are seldom used as outcome measures in exercise trials. A beneficial effect of exercise on fatigue has been reported for patients with different inflammatory rheumatic disease.^18^^,^^19^ However, previous research tended to focus on low-intensity flexibility exercises for patients with axSpA, and exercise intensity is crucial for the physiological effects.^16^ Hence, the potential effect of exercise on fatigue and mood in this patient group is not completely revealed.

Our research group recently completed a clinical trial that showed that high-intensity exercises reduced disease activity measured with the Ankylosing Spondylitis Disease Activity Score and the Bath Ankylosing Spondylitis Disease Activity Index, indicating that high-intensity exercises are safe for this patient group.^20^ Therefore, the aim of this secondary analysis was to investigate the effect of high-intensity cardiorespiratory and strength exercises on fatigue, mood, sleep quality, and perceived general health in patients with axSpA.

## Methods

### Design

This study was a secondary analysis of an assessor-blinded, 2-armed, multicenter, randomized controlled trial comparing the effects of 3 months of high-intensity exercises with no intervention (the Exercise for Spondyloarthritis [ESpA] study). Our study group have previously published a pilot study of this trial.^21^ The trial was conducted at rheumatology departments in Norway (Diakonhjemmet Hospital [DH], Martina Hansen Hospital [MHH] and the University Hospital of North Norway [UNN]) and in Sweden (Sahlgrenska University Hospital [SUH]). The study was approved by the Regional Committee for Medical and Health Research Ethics (REK South East 2015/86) in Norway and the Regional Ethical Review Board Gothenburg in Sweden (032–16). All procedures followed the Declaration of Helsinki, and all participants gave written and oral informed consent before entering. The study protocol is registered at ClinicalTrials.gov (NCT02356874).

### Participants

Patients were recruited from outpatient rheumatology departments at the 4 study centers as well as through various social media channels such as Facebook and websites for patients. The inclusion criteria were axSpA diagnosis (Assessment of SpondyloArthritis international Society criteria),^22^ age 18 to 70 years, no change in tumor necrosis factor inhibitor use during the previous 3 months, moderate to high disease activity (Bath Ankylosing Spondylitis Disease Activity Index ≥3.5)^23^ at prescreening, and not having performed regular cardiorespiratory or strength exercises >1 hour per week during the last 6 months. Exclusion criteria were established or symptoms of coronary heart disease (for details see Supplementary eFile 1), other comorbidity involving reduced exercise capacity (any contraindications/not able to exercise at a high intensity), inability to participate in weekly supervised exercise sessions, and pregnancy. Prescreening was administered by telephone.

### Exercise Intervention

The exercise program followed the American College of Sports Medicine exercise recommendations for cardiorespiratory and strength exercises and is described in detail in Supplementary eFile 2. Patients in the exercise group were encouraged to exercise 3 times a week for 3 months. Physical therapists specialized in rheumatology and trained in the exercise protocol supervised the exercises sessions twice a week at each study center. The supervised exercise sessions were organized as group sessions (7–12 participants) at the hospital at 2 of the study centers (UNN and SUH), whereas the physical therapist supervised the participants individually at a fitness center at the 2 other study centers (DH and MHH).

These supervised sessions consisted of 40 minutes of cardiorespiratory exercise and 20 minutes of strength exercises. The cardiorespiratory exercise was performed on a treadmill or a cycle ergometer at high intensity (10 minutes of warm-up, thereafter 4 minutes of forceful walking/running or bicycling at 90%–95% of maximal heart rate [HR] followed by 3 minutes of active resting at 70% of maximal HR repeated 4 times).^24^ Maximal HR was determined at baseline and was controlled by a Polar pulse-watch. The strength exercises were individually adapted to each participant according to the available equipment at each study center. The strength exercises focused on major muscle groups (squats, leg press, deadlifts, rows to chest, bench press, shoulder press, pulldowns, and sit-ups) and included 8 to 10 repetitions maximum in 2 to 3 sets. In addition to the supervised sessions, patients in the exercise group were encouraged to perform a cardiorespiratory exercise session once a week on their own for at least 40 minutes on intensity level above 70% of maximal HR (controlled by pulse-watch).

As a general rule, some pain (≤5 on a scale from 0–10) was tolerated during the exercises. However, if the pain got worse the day after, the exercises were adapted. Adaptations for pain were conducted on a regular basis, as it would have been done in a regular physical therapy practice.

### Control Group

Patients in the control group received no intervention and were asked to continue with their usual physical activity habits. All included patients received standard outpatient care from their respective hospitals, but before inclusion in the study it was specified that no change in medication before the 3-month assessment was desirable.

### Registration of Adherence and Safety

Exercise adherence was recorded by the physical therapist as attendance at the supervised sessions and as accomplishment of home sessions. The physical therapist checked the pulse watch to register fulfilment of home session every week. Exercise adherence was also self-reported as the participants in the exercise group recorded all sessions in a diary. The highest number of completed sessions reported by the physical therapist or self-reported in the diary was registered as total number of completed sessions. Patients in the control group did not record exercise session but were asked about exercise habits during the intervention period retrospectively in a questionnaire at the 3-month assessment.

Any adverse events that occurred during the supervised sessions or were reported from the participants from the home sessions were registered by the physical therapist who was in charge of the exercise program at each study center.

### Outcome Measures

All outcomes were secondary outcomes in the parent study.^20^ The primary outcome measure of the ESpA study was disease activity measured with the Bath Ankylosing Spondylitis Disease Activity Index^23^ and the Ankylosing Spondylitis Disease Activity Score.^25^ All outcomes were assessed by patient-reported questionnaire at baseline and immediately after the 3-month intervention. An assessor blinded for the participant’s group allocation administered the data collection and was available for the participants if they needed help with the questionnaires at the baseline- and 3-month follow-up assessment. After 12 months, a questionnaire was sent by postal mail to all participants together with a prepaid envelope.

Fatigue was assessed with the Fatigue Severity Scale (FSS).^26^^,^^27^ The FSS consists of 9 statements, each of which is scored from 1 to 7, where 1 indicates strongly disagree and 7 strongly agree. The mean of the 9 items yields the FSS score (0–7, 7 = worst). A cut-off value of ≥5 for severe fatigue was used.^27^ FSS is widely used and reported to have good concurrent validity with visual analog scale measures of fatigue (0.5–0.8). The FSS is also reported to have acceptable test-retest reliability (0.84)^26^ and has the ability to detect changes over time.^28^

Vitality was assessed with the vitality domain in the RAND 36-item Short form Health Survey (SF-36).^29^ The vitality domain in SF-36 consists of 9 questions regarding vitality during the last 4 weeks, each scored from 1 (all of the time) to 6 (not at all), and the mean of the 9 items yields a sum score from 0 to 100 (100 = best). The SF-36 as version 1 has been tested in rheumatoid arthritis patients, and it has shown good evidence for internal consistency and discriminant validity.^30^

Sleep quality was measured with the Pittsburgh Sleep Quality index (PSQI).^31^ The PSQI assesses sleep quality and disturbance over a 1-month time interval. It consists of 19 items that are grouped into 7 component scores, each weighted equally on a 0 to 3 scale, and the 7 components are summed to a global PSQI score (0–21, 21 = worst sleep quality). A cut-off score of >5 was used to categorize patients with reduced sleep quality.^31^ PSQI is widely used, reported to be valid as it discriminates patients with sleeping problems from controls, and it correlates to objective measures of sleeping quality measured by polysomnography.^31^^,^^32^ PSQI is also reported to have acceptable test-retest reliability.^31–33^

Mood was measured with the questions from the General Health Questionnaire 12 (GHQ-12).^34^ The GHQ-12 is a widely used and validated instrument that comprises 12 items reflecting several aspects of mood/depression.^34^ Each item was answered on a 4-point scale with the alternatives “not at all,” “no more than usual,” “rather more than usual,” and “much more than usual.” GHQ-12 gives a sum from 0 to 36 (36 = worst).

Perceived general health was assessed using the EuroQol applying a 0 to 100 visual analog scale where 0 = worst health and 100 = best health.^35^ Finally, background variables and information on disease-related variables were collected at baseline.

### Sample Size

The estimation of sample size was based on the primary outcome in the ESpA study,^20^ disease activity, and based on this we aimed at recruit 100 patients. Power calculations were not done for the outcome measures reported in this study.

### Randomization and Blinding

The randomization sequence was computer generated and stratified according to the study clusters, and a block randomization with a block size of 4 was used to ensure an adequate number of patients in the exercise group during the inclusion period. A secretary not involved in other aspects of the data collection prepared concealed envelopes that gave group allocation. The allocation was administered after baseline testing by the physical therapist who supervised the exercise sessions to keep the outcome assessor blinded for group allocation. Blinding of the participants and the physical therapists supervising the exercise sessions was not possible.

### Statistical Analyses

Data are shown as mean with SD, median with minimum and maximum values, or frequency with percentages. The statistical analyses were performed on the intention-to-treat population. Missing data were replaced by the last value carried forward approach. For continuous variables, analyses of covariance (ANCOVA) on the post-intervention values was used to assess the group differences with *P* values, mean difference, and 95% CI at 3- and 12-month follow-up. Baseline values and study center were included as covariates in the ANCOVA. We assessed the normality assumptions of the ANCOVA models by pp-plots of the residuals. To estimate effect sizes for continuous variables, the standardized mean difference with 95% CI was calculated using Review Manager 5.3 software. For categorical variables, groups were compared using logistic regression analysis. Poor sleep/severe fatigue was inserted as the dependent variable, study center was included as a covariate, and the results are given as odds ratios (ORs).

As the main analyses were performed on an intention-to-treat approach where missing values were imputed, a sensitivity analysis based on available data was also conducted. Statistical analyses were performed using SPSS version 21. *P* < .05 was considered statistically significant.

### Role of the Funding Source

The funder played no role in the design, conduct, or reporting of this study.

## Results

### Participants

**Figure 1:**
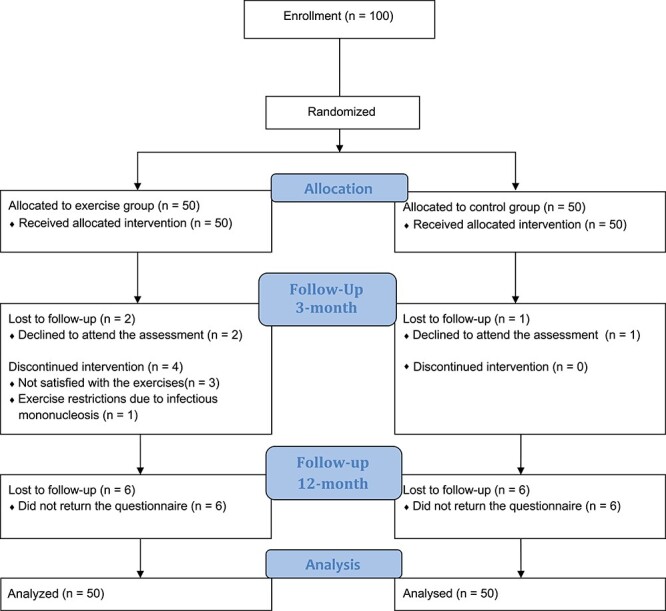
Flow of study participants.

A total of 100 patients with axSpA were included between August 2015 and September 2016. Fifty participants were allocated to the exercise group and 50 participants to the control group. The last 3-month assessments were performed in December 2016. Flow of participants throughout the trial is shown in Figure 1. Personal characteristics and disease-related variables at baseline are shown in Table 1.

**Table 1:** Baseline Descriptive of All Participants, Exercise Group, and Control Group*^a^*

	**All Participants, n = 100**	**Exercise Group, n = 50**	**Control Group, n = 50**
Age, y, mean (min-max)	46.2 (23–69)	45.1 (23–68)	47.2 (24–69)
Sex, male, n (%)	47 (47%)	25 (50%)	22 (44%)
≥13 years education	58 (58%)	29 (58%)	29 (58%)
Married/cohabitant	76 (76%)	39 (78%)	37 (74%)
In work, n (%)	81 (81%)	42 (78%)	39 (78%)
Current smoking, n (%)	12 (12%)	5 (7%)	7 (14%)
Medication			
NSAIDs, n (%)	71 (71%)	38 (76%)	33 (66%)
TNF inhibitor, n (%)	44 (44%)	22 (44%)	20 (40%)
Blood pressure medication	13 (13%)	8 (16%)	5 (10%)
Disease characteristics			
Radiographic axSpA	70 (70%)	38 (76%)	32 (64%)
Disease activity (ASDAS-CRP), mean (SD)	2.6 (0.7)	2.6 (0.8)	2.7 (0.6)
Disease activity (BASDAI), mean (SD)	5.1 (1.6)	4.9 (1.6)	5.3 (1.5)
CRP, median (min-max)	2 (2–28)	2 (2–28)	2 (2–13)
ESR (mm/h), median (min-max)	8 (1–67)	8 (2–67)	8 (1–28)
Physical function (BASFI), median (min-max)	3.2 (0.2–9.1)	2.6 (0.2–6.7)	3.0 (0.4–9.1)
Spinal flexibility (BASMI), mean (SD)	2.8 (1.3)	2.9 (1.3)	2.6 (1.3)
Fatigue measures			
Fatigue*^b^* (0–7, 7 = worst), mean (SD)	4.6 (1.3)	4.5 (1.2)	4.8 (1.3)
Severe fatigue (≥5), n (%)	57 (57%)	24 (48%)	33 (66%)
Vitality*^c^* (0,100, 100 = best)	56.1 (15.7)	59.6 (12.8)	53.6 (17.9)
Mood*^d^* (0–36, 0 = best), mean (SD)	16.6 (5.7)	16.3 (6.3)	16.9 (5.1)
Sleep quality*^e^* (0–21, 21 = worst), mean (SD)	9.1 (3.8)	9.2 (4.0)	9.1 (3.6)
Poor sleep (>5)	83 (83%)	42 (84%)	41 (83%)
Perceived general health*^f^* (0–100, 100 = best)	54.6 (17.0)	56.0 (14.0)	53.2 (19.6)

^a^ ASDAS = Ankylosing Spondylitis Disease Activity Score (higher score = worse); BASDAI = Bath Ankylosing Spondylitis Disease Activity Index; BASFI = Bath Ankylosing Spondylitis Functional Index; BASMI = Bath Ankylosing Spondylitis Metrology Index (All BAS instruments, 0–10, 10 = worst); CRP = C-reactive protein; ESR = Erythrocyte sedimentation rate; NSAIDs = non-steroidal anti-inflammatory drugs; TNF = tumor necrosis factor.

^b^ Fatigue severity scale.

^c^ RAND 36-item Short form Health Survey.

^d^ General Health Questionnaire.

*^e^* Pittsburgh Sleep Quality Index.

*^f^* EuroQol (0–100) VAS scale.

### Adherence and Safety

A total of 38 patients (76%) in the exercise group followed ≥80% of the prescribed exercise protocol (completed ≥29 of 36 sessions). Four (8%) patients quit the exercise program right after the starting date (for reasons, see Fig. 1). In general, based on reports from the physical therapist, all the participants met 90% to 95% of HR max during the intervals. However, 1 patient experienced chest pain and nausea during the exercises and completed the intervention at moderate intensity (64%–76% of HR max^16^) after advice from a cardiologist. No acute adverse events were reported during the exercise session, but 2 patients reported persistent pain during exercise. In the control group, 5 patients (10%) reported that they had performed cardiorespiratory or strength exercises ≥2 times a week during the 3-month intervention period.

At the 12-month follow-up, 43 participants in the exercise group reported their exercise habits and, at this time point, 17 of 43 (40%) reported to regularly perform strength and cardiorespiratory exercises.

### Effect of the Exercise Program at 3-Month Follow-up

**Figure 2:**
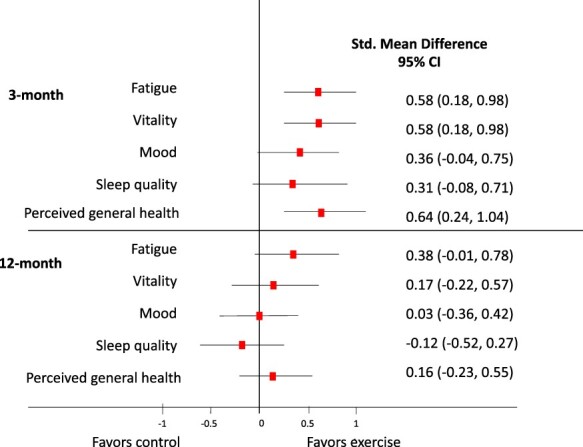
Forest plot of the effect of exercise on fatigue, vitality, mood, sleep quality, and perceived general health at 3- and 12-month follow-up. Data are shown as standardized mean difference (SMD, effect sizes) with 95% CI. SMDs between 0.2 and 0.4 are considered a small effect size, from 0.5 to 0.7 a medium effect size, and ≥0.8 a large effect size.

A significant beneficial effect of the intervention was seen on fatigue, vitality, and perceived general health and mood at the 3-month follow-up (Tab. 2). Furthermore, compared with the control group, the exercise group reduced the rate of having severe fatigue (OR = 0.41 [95% CI = 0.18–0.91], *P* = .03) and poor sleep quality (OR = 0.34 [95% CI = 0.13–0.92], *P* = .03) at the 3-month follow-up. There was no effect of the intervention on sleep quality analyzed as a continuous variable. As shown in the forest-plot in Figure 2, the effect sizes were medium for fatigue, vitality, and perceived general health, whereas it was small for mood and sleep quality.

**Table 2:** The Effect of Exercise on Fatigue, Vitality, Emotional Distress, Sleep Quality, and General Health at 3- and 12-Month Follow-Up

	**Exercise Group (n = 50)**			**Control Group (n = 50)**			**Effect 3 Mo**		**Effect 12 Mo**	
	**Baseline**	**3 mo**	**12 mo**	**Baseline**	**3 mo**	**12 mo**	**Effect (95% CI)** ^***a***^	***P***	**Effect** ^***f***^ ** (95%CI)**	***P***
Fatigue*^a^* (0–7, 7 = worst)	4.5 (1.2)	4.0 (1.2)	4.2 (1.3)	4.8 (1.2)	4.7 (1.2)	4.7 (1.3)	−0.42 (−0.7 to −0.1)	.01	−0.2 (−0.6 to 0.2)	.28
Vitality*^b^* (0–100, 100 = best)	59.1 (13.1)	66.8 (15.15)	61.4 (16.4)	53.5 (17.7)	57.6 (16.2)	58.4 (18.1)	5.0 (0.6 to 9.8)	.03	−0.3 (−6.2 to 5.5)	.91
Mood*^c^* (0–36, 0 = best)	16.3 (6.3)	13.9 (6.4)	15.8 (6.0)	16.9 (5.1)	16.0 (5.2)	15.6 (6.6)	−2.0 (−3.7 to −0.04)	.04	0.64 (−1.2 to 2.5)	.48
Sleep quality*^d^* (0–20, 20 = worst)	9.2 (4.0)	7.7 (4.1)	8.7 (4.5)	9.1 (3.6)	8.8 (3.3)	8.2 (3.5)	−1.1 (−2.1 to 0.2)	.10	0.52 (−0.82 to 1.87)	.44
General health*^e^* (0–100, 100 = best)	56.4 (14.1)	68.7 (15.9)	63.4 (17.1)	53.2 (19.6)	58.4 (16.0)	60.4 (20.5)	9.0 (3.3 to 14.7)	.002	2.5 (−4.8 to 9.7)	.50

***^a^* Values are shown as mean with SD, or differences with confidence intervals. :**Fatigue: the fatigue severity scale.

^b^ Vitality: Rand 36-item short form Health Survey.

^c^ Mood: the general health questionnaire.

^d^ Sleep quality with the Pittsburgh Sleep Quality Index.

^e^ Perceived general health with the EuroQol (0–100) VAS scale.

^f^ Estimated mean group difference, analyzed with ANCOVA with adjustments for baseline values and study center.

### Long-Term Effect of the Exercise Program

As expected, differences between the groups in fatigue, vitality, mood, sleep quality or perceived general health were not sustained at 12-month follow-up (Tab. 2). The medium to small effect sizes on the outcome measures seen at the 3-month follow-up were no longer present at the 12-month follow-up (Fig. 2). Furthermore, the number of patients reporting to have “severe fatigue” (OR = 0.32 [95% CI = 0.39–1.48], *P* = .67) or “poor sleep quality” (OR = 1.14 [95% CI = 0.46–2.83], *P* = .78) was similar in the groups at the 12-month follow-up.

The sensitivity analysis provided similar findings as the main analysis at the 3- and 12-month follow-up (data not shown).

## Discussion

This secondary analysis of the ESpA study showed that a 3-month high-intensity cardiorespiratory and strength exercise program had a beneficial effect on fatigue, vitality, and mood in patients with axSpA at the end of the intervention. Furthermore, participants in the exercise group had a lower OR for having poor sleep quality and severe fatigue than participants in the control group at the 3-month follow-up. There was no long-term effect of the exercise program. However, a long-term effect of exercise is not expected if the exercise is not maintained.^16^ The short-time beneficial effects showed in this study are important, as fatigue and sleep problems are 2 of the most frequently cited health aspects in which patients with axSpA would like to see an improvement.^2^^,^^3^ Indeed, it has been reported that up to 60% of patients with axSpA are affected by fatigue, and the prevalence of depression and anxiety is also high.^36^ AxSpA usually occurs in young adults, and therefore the negative effects of the disease on fatigue, mood, and vitality may impact patients’ social and working life through a considerable time span.^36^

To the best of our knowledge, the EspA study is the first large scale study investigating the effects of high intensity exercise in patients with axSpA. Previous exercise trials in patients with axSpA have mainly focused on flexibility exercises,^37^^,^^38^ and fatigue, sleep, and mood have seldom been used as outcome measures. Nevertheless, our findings are in line with the general perception that physical activity has a positive effect on sleep, mood, fatigue, vitality, and perceived health.^16^^,^^17^^,^^39^ Further, our result is consistent with a recent meta-analysis reporting a small beneficial effect of cardiorespiratory and strength exercises on fatigue in patients with inflammatory rheumatic diseases.^19^ The effect on fatigue was larger in the present study than in the meta-analysis, probably explained by higher exercise intensity.

In addition, our findings are also consistent with a randomized controlled trial reporting a significant beneficial effect of exercise on sleep in patients with rheumatoid arthritis.^40^ Although we found a small but non-significant effect on sleep measured with PSQI as a continuous variable, the improvement of a mean 1.0 point in PSQI in the exercise group was comparable with the result reported by Durcan et al.^40^ Hence, the difference in results between Durcan et al^40^ and the present study may be due to differences in baseline values and statistical analyses.

The mechanisms behind the observed positive effects on fatigue, sleep, mood, and perceived health may be explained by the beneficial effect of the intervention on inflammation, physical fitness, and pain,^20^ variables that are all closely related to our outcomes of interest. Firstly, the effect may be explained by the anti-inflammatory effect of exercise^41^ as inflammation is thought to play a role in the multifactorial development of disease symptoms such as fatigue. Secondly, the improvement in physical fitness increases the patients’ available reserve capacity and may thereby have beneficial effects on fatigue and vitality. Thirdly, the improvement in pain may have a positive impact on sleep as poor sleep in axSpA often is caused by pain in the second part of the night.^1^ In addition, the observed beneficial effects might have been caused by group interaction as the exercises were performed in a group setting for some of the participants. Hence, exercise has the potential to improve fatigue, sleep, and mood through various pathways, and the observed effect in the current study can probably be explained by an interaction of different mechanisms.

Even if small to moderate standardized effect sizes were found after intervention in the current study, the effect sizes were generally smaller than the reported Minimal Clinical Important Difference (MCID) values. For instance, the MCID for FSS is reported to be an effect size of 0.74 in patients with rheumatoid arthritis to move to a different fatigue category^42^ compared with 0.58 seen in our study, and for the SF-36 vitality score the MCID is −10.7 (95% CI = 5.9–15.5)^42^ compared with 5 points in our study.

As expected, no long-term effects of the exercise program were seen on any of the secondary outcomes, as effects of exercising are dependent on regular participation. Exercise-induced adaptations are reversed over time without adherence to the exercise program, and it is difficult for people to adhere to physical activity at a high intensity level.^16^ In the present trial, no follow-up or monitoring was provided after the end of the 3-month program. At the 12-month follow-up, less than one-half of the participants in the exercise group reported regularly performing cardiorespiratory and strength exercises. Hence, we therefore did not expect effect of the exercise program on fatigue, sleep, or mood 9 months after the end of the program.

Fatigue and lack of energy is frequently reported as a barrier for being physically active,^43^^,^^44^ and it is easy to imagine that the feeling of being exhausted particularly is a barrier for participation in high-intensity exercise. In the same way, energy conservation has traditionally been recommended by health personnel in the management of fatigue and chronic pain.^45^ Although there is a general perception that graded aerobic exercise has a beneficial effect on chronic fatigue,^6^ there is a gap between graded aerobic exercise and high-intensity exercise like the intervention in the present study. The finding of a positive effect on fatigue and vitality after high-intensity exercises is interesting and should be taken into account when considering energy conservation in the management of fatigue. In addition, since pharmacological treatment has failed to show an effect on fatigue,^10^ it can be argued that high-intensity exercise is an important therapeutic tool in the management of fatigue in patients with axSpA.

Strengths of the current randomized controlled trial include the study design and the pragmatic approach. The intervention was delivered by a local physical therapist at 4 different centers, which increases the generalizability of the results. Due to the demanding exercise program, we may have recruited motivated patients, which can have biased the results. Nevertheless, the majority of the patients were recruited from outpatient clinics, and almost one-half of them were treated with tumor necrosis factor inhibitors, thus strengthening the generalizability of the results. Further, the exercise program followed generic exercise guidelines, the adherence to the exercise protocol was high, and losses to follow-up were very low.

The main limitation with the present study is the lack of blinding of participants, which is not possible when comparing exercise with no intervention. Further, a limitation is that all the outcomes are secondary outcomes of the randomized controlled trial, and therefore we cannot rule out a problem with multiple testing. However, the results were consistent, showing a beneficial effect of the exercise program on all secondary outcomes, which limits the risk of statistical chance. Lack of blinding of participants represents a risk of bias^46^ as all the outcome measures were self-reported. Despite this, beneficial effects of the intervention on objective outcomes such as physical fitness and C-reactive protein^20^ strengthen the validity of the results. Adverse events during the intervention period were only registered in the exercise group, and the groups therefore cannot be compared regarding this issue. Finally, 3 participants in the exercise group discontinued the intervention because they were not satisfied with the exercises, hence indicating that high-intensity exercise is not feasible for all individuals with axSpA.

In conclusion, this is the first large-scale study, to our knowledge, to demonstrate that a high-intensity cardiorespiratory and strength exercise program has a short-term beneficial effect on fatigue, vitality, sleep, and mood in patients with axSpA, but, as expected, long-term effects were not seen. High-intensity exercises should therefore be recommended as a part of the management, and strategies for long-term exercise adherence should be developed.

## Supplementary Material

SupplementaryFile1_pzaa086

SupplementaryFile_2_pzaa086

## Clinical Trial Registration

The study protocol is registered at ClinicalTrials.gov (NCT02356874).
