# Supplementary material for: High-Intensity Exercise Improves Fatigue, Sleep, and Mood in Patients With Axial Spondyloarthritis: Secondary Analysis of a Randomized Controlled Trial
Source: Phys Ther. 2020 May 4;100(8):1323–32. doi: 10.1093/ptj/pzaa086 (PMC7439225; doi:10.1093/ptj/pzaa086)
Supplement: SupplementaryFile1_pzaa086 [file supplementaryfile1_pzaa086.docx]

| **Supplementary file 1 Screening of cardiovascular diseases** |
| --- |
| 1. **Patients with cerebral stroke, established coronary heart disease (prior myocardial infarction or angina pectoris) and or an indication of ischemic heart disease will be excluded from the ESpA-study.** |
| 1. **Screening questions at baseline:**  - Relatives who have died suddenly and unexpected before the age of 40 years. - Indication of ischemic heart disease during physical activity such as chest pain, dizziness or abnormal dyspnea - First-degree relatives diagnosed with hypertrophic cardiomyopathy - ECG signs of ischemic heart disease shown at a previous consultation |
| 1. **Blood pressure (measured at baseline)**   Systolic blood pressure > 200 mmHg or diastolic blood pressure > 115 is a contraindication for physical activity, and participants are excluded from the study. |
| 1. **Atrial fibrillation (measured at baseline)**   If atrial fibrillation is detected at baseline the patients is excluded from the ESpA-study. |

Reprinted with permission, first published in Sveaas et al. Br J Sports Med. 2019: doi:10.1136/bjsports-2018-099943.
