# Supplementary material for: High-Intensity Exercise Improves Fatigue, Sleep, and Mood in Patients With Axial Spondyloarthritis: Secondary Analysis of a Randomized Controlled Trial
Source: Phys Ther. 2020 May 4;100(8):1323–32. doi: 10.1093/ptj/pzaa086 (PMC7439225; doi:10.1093/ptj/pzaa086)
Supplement: SupplementaryFile_2_pzaa086 [file supplementaryfile_2_pzaa086.doc]

| **Supplementary file 2 Exercise program the ESpA-(exercise for spondyloarthritis)-study**  Exercise period: 3 months | |
| --- | --- |
| **Cardiorespiratory exercises (3 days a week)** -HR_max_ was determined at the end of a maximal treadmill test. | |
| **High Intensity interval exercise^14^** | |
| Delivery  Type  Frequency  Intensity  Time | Supervised by a physiotherapist at a gym in a hospital or a fitness centre  Walking or running on a treadmill/cycle ergometer  2 days per week  10 min warm up at 70% of HR_max_. 4 x 4 min interval exercise at 90-95% of HR_max_ with 3 min of active resting period at 70% of HR_max_ between each interval. 3 min cool down at 70%. The intensity was controlled by a Polar puls watch during each session.  38 minutes |
| **Home session** | |
| Delivery Type  Frequency  Intensity  Time | Unsupervised individual training  Walking/running/cycling outdoor or at fitness center  1 day per week  ≥70% of HR_max_. The intensity was controlled by a Polar puls watch.  ≥40 minutes |
| **Muscular strength exercises (2 days a week)-** Started with 2-3 weeks with gradually adaption before the work load was set to 8-10 repetitions maximum | |
| Delivery  Type  Pattern  Frequency  Intensity  Time  Repetitions  Sets  Progression | Supervised by a physiotherapist (performed after the high intensity interval exercise)  Six exercises for major muscle groups, individually adapted. Preferably with external load. Examples of exercises: Squat, leg press, deadlifts, rows to chest, bench press, shoulder press, pull downs and sit-ups.  Circle of exercises or switching between two exercises (no rest-intervals).  2 days per week  8-10 repetitions maximum  20 minutes  8-10 repetitions  2-3 sets  If the patient could perform more than 10 repetitions per sets, the workload was increased |
| The exercise program followed the American College of Sports Medicine exercise recommendations.  HR; Heart rate | |

Reprinted with permission, first published in Sveaas et al. Br J Sports Med. 2019: doi:10.1136/bjsports-2018-099943.
